# Case of Chronic Dyssentery of Three Years Duration, Cured by the Blue Pill, and a Mucilaginous Diet

**Published:** 1830

**Authors:** R. Bard

**Affiliations:** N. Troy, Vermont


Art. IV.—*Case of Chronic Dysentery of three years duration, cured by the blue pill and a mucilaginous diet,*—by D. R. Bard, M. D. of N. Troy, Vermont.

In the month of September last, I was requested to prescribe for a young married lady who had been labouring under chronic dysentery, for three years, with but slight and partial remissions in the winter months. She was much emaciated; pulse quick and feeble; countenance sallow; skin dry and harsh; tongue slightly furred; appetite very bad, great debility. There were generally from 10 to 15 dejections daily, slimy or purulent, streaked with blood, and sometimes pure blood was discharged. The abdomen was tender on pressure, with great pain and weakness in the loins, sacrum and hips, and the evacuation of the urine and fæces appeared to be prevented by a peculiar painful sensation of muscular debility distinct from the tenesmus. As the patient had been already under the care of numerous practitioners, who had tried almost every variety of treatment without success. I determined to resort to the practice recommended by Dr. Mitchell, in the Journal of the Medical & Physical Sciences for August, 1828, viz: a mucilaginous diet with the blue pill, and such occasional depletion as the symptoms might call for.

On the 3d of September, the patient was directed after evacuating the bowels by a dose of castor oil, to take nothing into the stomach but an infusion of slippery elm, and 4 grs. of the blue pill every night.

4th. The oil operated well; two evacuations since.

6th. Feels very feeble and faint; tongue clean, pulse softer, skin more pliant; has had three stools both yesterday and to day, similar in character, but with less tenesmus. Treatment continued.

9th. One stool daily since the last report, improved in appearance and without tenesmus, distress in the pelvis abated, and the tenderness of the abdomen removed.

12th. On the 10th she had a slight return of the pain and bloody discharges, occasioned probably, by drinking a cup of tea and eating a biscuit. Since that time, having confined herself to the infusion of elm, the symptoms have improved, and she has had one consistent evacuation daily.

15th. All her difficulties appear to be removed. Permitted to use a little stale bread and milk.

She continued to use the blue pill and the infusion of elm for sometime, and gradually resumed her former diet without inconvenience, and has since enjoyed perfect health.

Although the pathology of chronic dysentery is perhaps as well settled, as that of any other disease, there are few practitioners who have not occasionally found their best concerted plans of cure frustrated.

The influence of a mucilaginous diet, by removing irritation from the debilitated digestive organs and their ulcerated surfaces, can be readily comprehended. The *modus operandi* of the blue pill is not so obvious. If, as Dr. Mitchell supposes, it is essential to the cure, does its efficacy arise from its power in correcting derangements of the digestive organs, or from its local application to the diseased surfaces? If its utility depends upon its general effects as a mercurial, could not some other preparation of mercury supply its place? These enquiries are proposed to those persons whom, a more extended acquaintance with the disease may qualify to solve them, better than can possibly be the case with the practitioner, whose practice is confined to a high northern latitude.

